# Development of the Diabetic Kidney Disease Mouse Model Culturing Embryos in α-Minimum Essential Medium *In Vitro*, and Feeding Barley Diet Attenuated the Pathology

**DOI:** 10.3389/fendo.2021.746838

**Published:** 2021-11-02

**Authors:** Shiori Ishiyama, Mayu Kimura, Takao Nakagawa, Yuka Fujimoto, Kohei Uchimura, Satoshi Kishigami, Kazuki Mochizuki

**Affiliations:** ^1^ Department of Integrated Applied Life Science, Integrated Graduate School of Medicine, Engineering, and Agricultural Sciences, University of Yamanashi, Kofu, Japan; ^2^ Kiwa Laboratory Animals Co., Ltd., Kiminocho, Japan; ^3^ Advanced Biotechnology Center, University of Yamanashi, Kofu, Japan; ^4^ Division of Nephrology, Department of Internal Medicine, Interdisciplinary Graduate School of Medicine and Engineering, University of Yamanashi, Kofu, Japan; ^5^ Faculty of Life and Environmental Sciences, University of Yamanashi, Kofu, Japan

**Keywords:** diabetic kidney disease (DKD), MEM mice, DOHaD (developmental origins of health and disease), barley, glomerulosclerosis, transforming growth factor beta (TGF- β)

## Abstract

Diabetic kidney disease (DKD) is a critical complication associated with diabetes; however, there are only a few animal models that can be used to explore its pathogenesis. In the present study, we established a mouse model of DKD using a technique based on the Developmental Origins of Health and Disease theory, i.e., by manipulating the embryonic environment, and investigated whether a dietary intervention could ameliorate the model’s pathology. Two-cell embryos were cultured *in vitro* in α-minimum essential medium (MEM; MEM mice) or in standard potassium simplex-optimized medium (KSOM) as controls (KSOM mice) for 48 h, and the embryos were reintroduced into the mothers. The MEM and KSOM mice born were fed a high-fat, high-sugar diet for 58 days after they were 8 weeks old. Subsequently, half of the MEM mice and all KSOM mice were fed a diet containing rice powder (control diet), and the remaining MEM mice were fed a diet containing barley powder (barley diet) for 10 weeks. Glomerulosclerosis and pancreatic exhaustion were observed in MEM mice, but not in control KSOM mice. Renal arteriolar changes, including intimal thickening and increase in the rate of hyalinosis, were more pronounced in MEM mice fed a control diet than in KSOM mice. Immunostaining showed the higher expression of transforming growth factor beta (TGFB) in the proximal/distal renal tubules of MEM mice fed a control diet than in those of KSOM mice. Pathologies, such as glomerulosclerosis, renal arteriolar changes, and higher TGFB expression, were ameliorated by barley diet intake in MEM mice. These findings suggested that the MEM mouse is an effective DKD animal model that shows glomerulosclerosis and renal arteriolar changes, and barley intake can improve these pathologies in MEM mice.

## Introduction

Type 2 diabetes mellitus (T2DM) is a major cause of chronic kidney disease (1, 2), commonly known as diabetic kidney disease (DKD). DKD generally develops and progresses with renal glomerular hyperfiltration, microalbuminuria, apparent albuminuria, and low glomerular filtration rate (GFR), and patients eventually require dialysis (3). A serial, cross-sectional Japanese T2DM cohort study reported that the number of patients with DKD with an estimated glomerular filtration rate (eGFR) < 60 mL/min/1.73 m^2^ increased from 12.1% in 1996 to 24.0% in 2004 (4). Therefore, the mechanistic exploration of DKD development and therapy are necessary for DKD prevention.

Glomerular and tubular disorders, induced by mesangial matrix expansion, are considered to be the main features of DKD. Hyperglycemia is known to promote the proliferation of mesangial cells, which leads to the excess production of extracellular matrix (ECM) and induces glomerulosclerosis (5, 6). In addition, hyperglycemia induces the migration of pericytes from the peritubular capillaries to the interstitial space, thereby causing arteriolosclerosis (7). Furthermore, the migration of peritubular pericytes accelerates tubular interstitial changes by enhancing the transition of pericytes into myofibroblasts. DKD can be classified into four categories based on the type of hierarchical glomerular lesions, and tuberous sclerosis is the most characteristic lesion in DKD, with extensive interstitial and vascular lesions formed in DKD of each category (8). Glomerular cell dysfunction impairs glomerular filtration and microvascular permeability, which reduces the levels of body wastes (such as nephrotoxins) in the urine, and also reduces microalbuminuria or albuminuria. However, dietary or drug therapies to prevent or ameliorate DKD are not well developed.

The lack of animal models with disease development similar to that occurring in patients with DKD has delayed research and development of DKD therapies. Several T2DM animal models, such as mutant T2DM models and spontaneous T2DM models, as well as conventional models, exhibit DKD. Compared with age-matched non-diabetic *db*/*m* control mice, obese diabetic *db*/*db* mice carrying the mutant leptin (an anorexigenic hormone) receptor exhibited six times higher urinary albumin levels and lower GFRs at 28 weeks of age and greater mesangial matrix expansion after 16 weeks of age (9–11). *db*/*db* mice with unilateral renal artery stenosis developed severe mesangial sclerosis, progressive interstitial fibrosis, tubular atrophy, and interstitial inflammation, but not mesangial matrix expansion, a major characteristic of DKD (12). Compared with lean control rats, the T2DM model Zucker rat, which carries a mutant leptin receptor and develops obesity at younger ages, exhibited 200-fold higher urinary albumin levels at 16 weeks and 1000 times higher hyperfiltration (non-decreased 50% eGFR) at 26 weeks. These phenotypes can be considered severely pathological compared with those of patients with DKD (13). OLETF rats, which are spontaneous T2DM model rats with a lack of cholecystokinin 1 receptor gene, exhibited diffuse glomerular sclerosis and tuberous sclerosis, along with basement membrane thickening, mesangial proliferation, and fibrin cap formation (14). However, compared with control OLETF rats, OLETF rats fed a 40% (w/w) high-protein diet from 5 to 30 weeks of age showed a progression in nephropathy at 30 weeks, even though they exhibited relatively lower blood glucose levels in the oral glucose tolerance test (OGTT) than 26-week-old control OLETF rats (15). Therefore, diabetes cannot be considered the main cause of kidney dysfunction in the OLETF model. In addition, because T2DM and DKD development in OLETF rats takes longer (20 or more weeks of age) than that in *db*/*db* mice (18 weeks of age), and the developmental stages vary widely among different experimental groups, the OLETF rat model is not suitable for studies on DKD (11, 15). Therefore, the animal models mentioned above do not replicate the manifestations of DKD observed in patients, such as middle albuminuria, glomerular hypertrophy, and mesangial matrix expansion (16).

Recent studies have suggested that environmental factors during developmental stages can induce metabolic diseases, including T2DM and DKD. This is stated as the Developmental Origins of Health and Disease (DOHaD) theory. A retrospective cohort study in Ukraine reported that the odds ratio of T2DM diagnosis at age 40 years or older was higher in individuals born in areas affected with severe-to-extreme famine than in individuals born during famine but in unaffected areas (17). Furthermore, it was reported that the body weight as per gestational age shows positive correlation with total kidney volume at 0, 3, and 18 months after birth. Additionally, premature (< 37 weeks of gestation) children had smaller kidneys compared to mature children (37 to 42 weeks of gestation) (18). In a retrospective case-control study of infants born at ≤ 34 weeks of gestation, compared with appropriate for gestational age premature infants, small for gestational age premature infants had higher serum creatinine on postnatal days 1 and 3 and a lower urinary output (in mL/kg/h) (19). These findings suggest that undernutrition during the gestational period is a risk factor for the development of T2DM and renal insufficiency in adulthood, and SGA offspring are predisposed to these conditions. Recently, we established a T2DM mouse model using techniques based on the DOHaD theory, such as manipulation of the embryonic environment and subsequent administration of high-fat, high-sugar diets. Specifically, we established a mouse model using two-cell-stage embryos cultured in α-minimal essential medium (αMEM), followed by embryo transfer into the mother (MEM mice). After birth, the mice were fed a high-fat, high-sugar diet after weaning, and hence, were remarkably hyperglycemic and moderately overweight, similar to patients with T2DM, particularly Asian patients (20). MEM mice also developed non-alcoholic hepatic steatosis with hepatic fibrosis (21), which is frequently observed in patients with T2DM. The factors influencing T2DM development in MEM mice, such as the environmental conditions during the fetal and postnatal periods, is similar to those in patients with T2DM. In addition, the intake of barley, a food abundant in the soluble dietary fiber β-glucan, reduced postprandial hyperglycemia (22) and repressed hepatic fibrosis in MEM mice. However, it is unclear whether MEM mice develop DKD, and whether dietary factors, including barley, attenuate DKD in MEM mice.

In this exploratory animal study, we investigated whether T2DM MEM mice develop DKD, and whether barley intake after birth alleviates the pathology.

## Materials And Methods

### Animals

We have previously demonstrated that mice developed from embryos cultured *in vitro* in α-MEM (MEM mice) exhibit T2DM with postprandial hyperglycemia and non-alcoholic steatohepatitis, in contrast to mice developed from embryos cultured *in vitro* in potassium simplex optimized medium (KSOM) (23). Barley intake for 10 weeks ameliorated non-alcoholic steatohepatitis in MEM mice (21). In this study, we used the same mice (MEM mice and KSOM mice) to explore whether MEM mice develop DKD and to investigate the effects of barley intake on renal pathology. Briefly, 2-cell embryos were obtained from the uteri of Institute of Cancer Research (ICR) pregnant mice aged 8 weeks, and subsequently, the 2-cell embryos were cultured in either α-MEM (135-15175, Wako Pure Chemical Industries, Ltd., Osaka, Japan) or KSOM (ARK Resource, Kumamoto, Japan) control medium (Table S1) for 48 h (morula stage) at 37 °C in a 5% CO_2_ incubator. Subsequently, to develop MEM or KSOM mice, the morulae were transplanted in another pregnant mouse (aged 8 weeks) and pregnant mothers, and the mothers with suckling pups were fed the laboratory chow diet (MF, Oriental Yeast Co., Ltd., Tokyo, Japan) until weaning (21 days) at Kiwa Laboratory Animal Co., Ltd. (Wakayama, Japan). After weaning, the pups were fed the laboratory chow diet until they were of 8 weeks, and subsequently, they were fed a high-fat, high-sugar (Western-style) diet (**Supplementary Table S2**) for 58 days. At age 19–25 weeks, MEM/ICR (n = 24) and KSOM/ICR male mice (n = 8) were moved to the University of Yamanashi, where they were provided water and food *ad libitum*, placed in cages (two per cage), and maintained under controlled conditions (temperature 23 ± 2°C; humidity 50% ± 10%; 12 h light/12 h dark cycle). MEM mice were then randomly allocated to two groups of similar age and body mass. Thus, three groups were formed: MEM mice fed a rice-based diet (Niigata Flour Milling Co., Ltd., Niigata, Japan; n = 12; MR group), MEM mice fed a diet containing barley powder (Hakubaku Co., Ltd., Yamanashi, Japan; n = 12; MB group), and KSOM control mice (n = 8) fed a rice-based diet (KC group). One animal in the MB group died during the OGTT and, therefore, its data was not included in the experimental data (21, 23). The diet composition provided by Oriental Yeast Co., Ltd. is provided in **Supplementary Table S2**. We did not calculate the sample sizes or perform the study under blinded conditions because this was an exploratory study. The β-glucan content in the barley diet was 1.06 g/100 g barley (average; n = 2), as determined at Hakubaku Co., Ltd. This animal study was approved by the Ethics Committee of the University of Yamanashi (approval number A30-24) and was performed according to the institutional animal experiment guidelines. The mice were decapitated, and samples were collected from one mouse at a time in the order of MR, MB, and KC to ensure that each group had similar mean dissection times (9:00 am–3:00 pm) (21, 23). Kidney tissue samples were collected and weighed, and the right kidney tissues were snap-frozen in liquid nitrogen and stored at −80°C until use for qRT-PCR and western blotting.

### Histological Staining of the Pancreas and Kidney Sections

The pancreas and left kidney were divided into three equal parts, and the middle sections were immediately fixed with 4% paraformaldehyde and incubated overnight in phosphate-buffered saline, with the solution switched to 70% ethanol prior to processing for paraffin embedding, as described previously (21). Each tissue section was embedded in paraffin by New Histo. Science Laboratory Co., Ltd. (Tokyo, Japan). The pancreas sections were stained with hematoxylin-eosin (HE) stain and Masson’s trichrome (MT) stain for quantifying the islets of Langerhans and pancreatic β cells and estimating fibrosis. The kidney sections were subjected to Periodic Acid-Schiff (PAS) and Elastica van Gieson (EVG) staining at KAC Co., Ltd. (Shiga, Japan) for quantification of mesangial expansion, glomerulosclerosis, and renal artery injury. Immunostaining for insulin (rabbit monoclonal antibody, 1:1,000; #3014, Cell Signaling Technology) and TGFB (transforming growth factor beta; rabbit polyclonal antibody, 1:1,000; #3711, Cell Signaling Technology) was performed at KAC Co., Ltd. Subsequently, the islets of Langerhans of the pancreas, and the glomerulus, tubule, and renal artery were examined under a light microscope (CX41LF, Olympus Corp., Tokyo, Japan). The islets of Langerhans were observed using HE staining. The fibrotic and insulin-positive areas as well as the pancreatic β cells were analyzed from the digital images (five images per a mouse). Glomerular expansion was assessed using the fractional average diameter based on ten glomeruli per a mouse, and glomerular fibrosis was defined based on the mesangial matrix area (PAS-positive area) per unit diameter, based on observations in ten glomeruli per mouse. The nodular lesion ratios were quantified by counting the pathological, altered glomeruli per total glomeruli in each specimen. Intimal thickening of the renal artery was determined as a percentage of the outer diameter (OD) (%OD), as described previously (24). In brief, %OD = 100(T+S)/2OD, where (T+S)/2 is the average of the two-sided intimal thickness. Arteriolar intimal hyalinosis was observed, as reported in previous studies (24, 25), and the hyalinization ratio was quantified in terms of hyalinization vessel counts per total vessel counts in each specimen. The TGFB-positive area was measured in the glomeruli, proximal tubule, and distal tubule, as described previously (26) (one image was randomly selected per a mouse, n = 8–12 images in each experimental group). All digital images were analyzed using the ImageJ software (Image Processing and Analysis in Java, NIH, Bethesda, MD, USA), as recommended (27).

### Preparation of Kidney Homogenates and Biochemical Analysis

Approximately 100 mg of each frozen kidney sample was homogenized in 1 mL of RIPA buffer (1% NP-40, 0.1% sodium dodecyl sulfate, 20 mM Tris-HCl [pH 8.0], 5 mM EDTA, 150 mM NaCl, 1 mM Na_3_VO_4_, 0.1 mM Na_2_MoO_4_, and 10 mM NaF) containing protease inhibitor cocktail tablets (cOmplete™, Roche Diagnostics K.K., Risch-Rotkreuz, Switzerland), as described previously (21, 23). Five hundred microliters of the homogenates were dispensed and used to measure the levels of malondialdehyde (MDA), 8-hydroxydeoxyguanosine (8-OHdG), and other oxidative markers, in the kidney, as well as for western blotting. Blood glucose and insulin concentrations were measured as described previously (21, 23). Renal and urinary 8-OHdG levels were measured using a highly sensitive ELISA kit for 8-OHdG (Japan Institute for the Control of Aging NIKKEN SEIL CO, Ltd., Shizuoka, Japan), and the renal MDA concentration was measured using a NWLSS™ Malondialdehyde Assay (Northwest Life Science Specialties, LLC, Vancouver, WA, USA). The phosphorous content in the collected plasma samples was measured using a phospha-C Test Wako kit (FUJIFILM Wako Pure Chemical Corporation, Osaka, Japan). All tests were performed according to the manufacturer’s instructions.

### Statistics Analysis

The results are expressed as mean ± standard error of the mean (SEM). In this study, there were two explanatory variables, such as the difference in culture medium (MEM and KSOM) for *in vitro* embryos and diet differences from the adult stage (control diet and barley-based diet). Therefore, we used Student’s *t*-test to compare each explanatory variable between the MEM and KSOM groups or between the control and barley-based diet groups. A *P* value < 0.05 was considered to be statistically significant. All values were analyzed using Excel Statistics 2010 (Social Survey Research Information Co., Ltd., Tokyo, Japan).

## Results

### Characteristics and Biochemical Parameters of MEM Mice

As described previously (21, 23), the body weight, non-fasting blood glucose concentrations, and insulin concentrations in MR mice were not higher than those in KC or MB mice. However, food intake was higher in MR mice than in KC mice, and the weight of the pancreas per unit body weight was lower in MB mice than in MR mice. In this study, we measured the weights of the pancreas and kidneys; MDA and 8-OHdG concentrations in the kidney and urine; and plasma phosphorus concentrations in mice. The weights of the kidney and pancreatic tissues did not differ between MR and KC mice or between MR and MB mice. The renal concentration of MDA, an oxidative stress marker, was lower in MB mice than in MR mice, but did not differ between MR and KC mice. The concentrations of 8-OHdG, an indicator of oxidative stress, in the kidney and urine, did not differ between MR and KC mice or between MR and MB mice. MR mice showed higher plasma phosphorus concentrations than KC mice, whereas MB mice showed lower plasma phosphorus concentrations than MR mice (*P* = 0.055) (**Table 1**). The urinary albumin was not detected (data not shown).

**Table 1:** Metabolic variables of KSOM control mice and MEM mice after 10 weeks of control or barley diet feeding.

	KC	MR	MB
Age (weeks)	28 ± 0.0	30 ± 0.6*	30 ± 0.7
Body weight (g)	70 ± 3.5	81 ± 4.2	84 ± 3.8
Blood glucose (mg/dL)	191 ± 7	334 ± 73	290 ± 71
Blood insulin (mg/dL)	3.3 ± 1.2	2.3 ± 0.6	1.1 ± 0.1
Kidney weight/body weight (g/g)	0.013 ± 0.001	0.014 ± 0.002	0.013 ± 0.002
Pancreas weight/body weight (g/g)	0.008 ± 0.000	0.009 ± 0.001	0.007 ± 0.001^#^
Plasma phosphorus (mg/dL)	7.19 ± 0.23	8.15 ± 0.32*	7.32 ± 0.24
Renal MDA/protein (μM)	6.1 ± 0.2	6.7 ± 0.7	5.4 ± 0.5^#^
Renal 8-OHdG (ng/mL)	0.87 ± 0.04	1.26 ± 0.18	1.07 ± 0.04
Urinary 8-OHdG (ng/mL)	0.03 ± 0.02	0.10 ± 0.03	0.17 ± 0.10
Food consumption (g/day)	4.74 ± 0.04	7.22 ± 0.46*	8.56 ± 1.08

8-OHdG, 8-hydroxydeoxyguanosine; MB, minimum essential medium (MEM) mice on a barley diet; MDA, malondialdehyde; MR, MEM mice on a rice diet; KC, potassium simplex optimized medium control mice on a rice diet. Data are expressed as means ± SEM for 8-12 mice. Statistical analyses were performed by Student’s t-test. *P < 0.05 compared with the KC group; ^#^P < 0.05 compared with the MR group.

### Pancreatic Exhaustion in MEM Mice Under Diabetic Conditions

**Figure 1:**
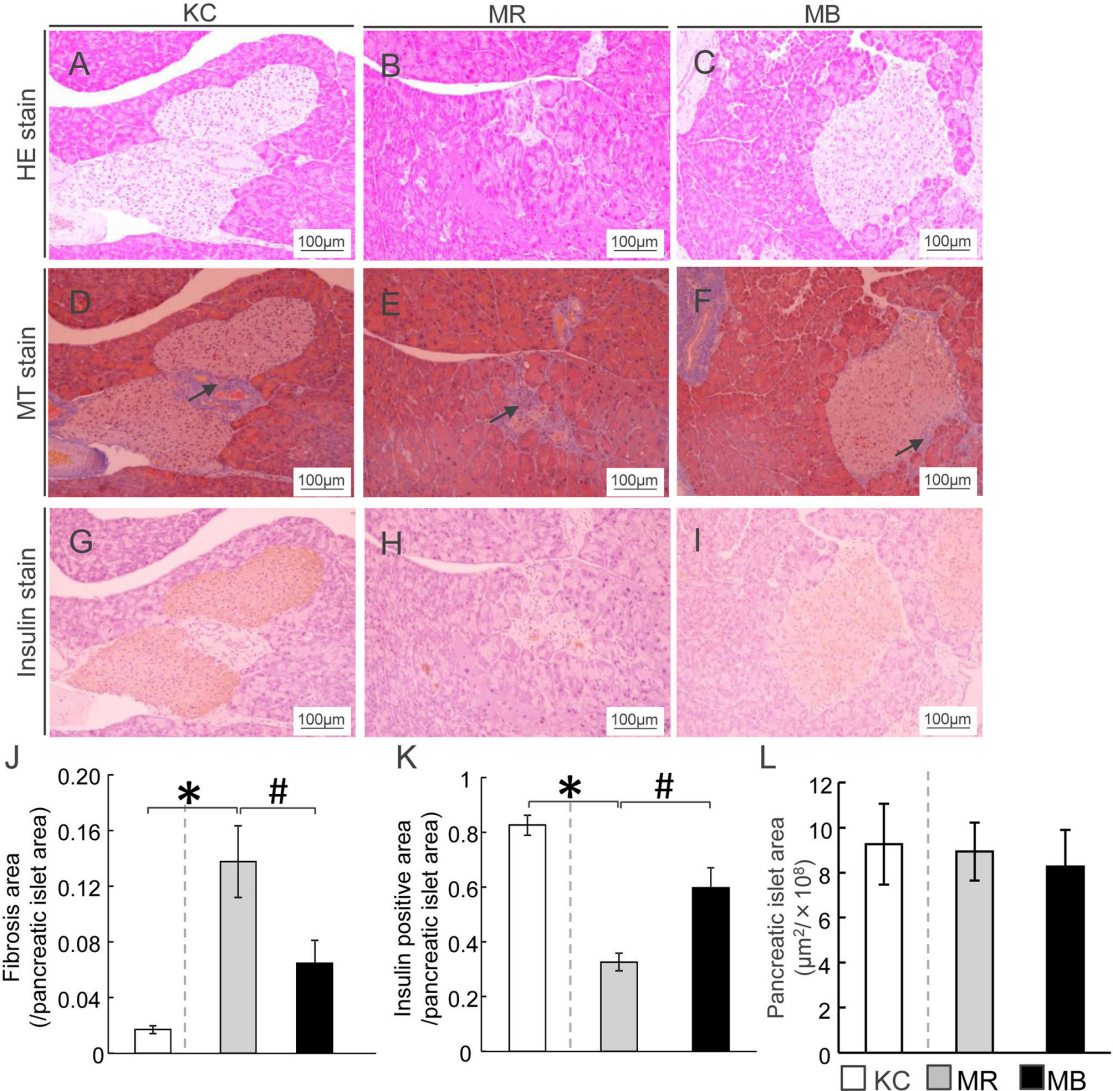
β-Cell area in mice after 10 weeks of experimental diet intake. **(A–C)** Representative histological hematoxylin-eosin staining of pancreatic sections from mice from each of the three experimental groups. **(D–F)** Fibrotic areas in the pancreas within the islets were stained blue after Masson’s trichrome staining (arrows). **(G–I)** Insulin-positive areas within the islets are stained brown (scale bar, 100 µm). Ratio of areas with fibrosis **(J)** and insulin-positive cells **(K)** in the pancreatic islets and area of pancreatic islets **(L)**. MB, minimum essential medium (MEM) mice on a barley diet; MR, MEM mice on a rice diet; KC, potassium simplex optimized medium control mice on a rice diet. Data are expressed as the mean ± SEM for 8–12 animals. Data were analyzed using Student’s *t*-test. *P < 0.05 compared with the KC group; ^#^ *P* < 0.05 compared with the MR group.

The islets of Langerhans were assessed by HE-staining, and the areas of fibrosis, and insulin positivity and pancreatic β cells in each islet of Langerhans was assessed by MT staining and immunostaining for insulin, respectively (**Figures 1A–I**). The fibrotic areas were larger in MR mice than in KC mice and smaller in MB mice than in MR mice (**Figure 1J**). The insulin-positive area was smaller in MR mice than in KC mice, whereas it was larger in MB mice than in MR mice (**Figure 1K**). The islet size did not differ between MR mice and KC mice or MR mice and MB mice (**Figure 1L**). All images of HE, MT and Insulin staining of the pancreas of each group (**Supplementary Figures S1–S3**).

### Increased Number of Glomerular Lesions in Diabetic MEM Mice

**Figure 2:**
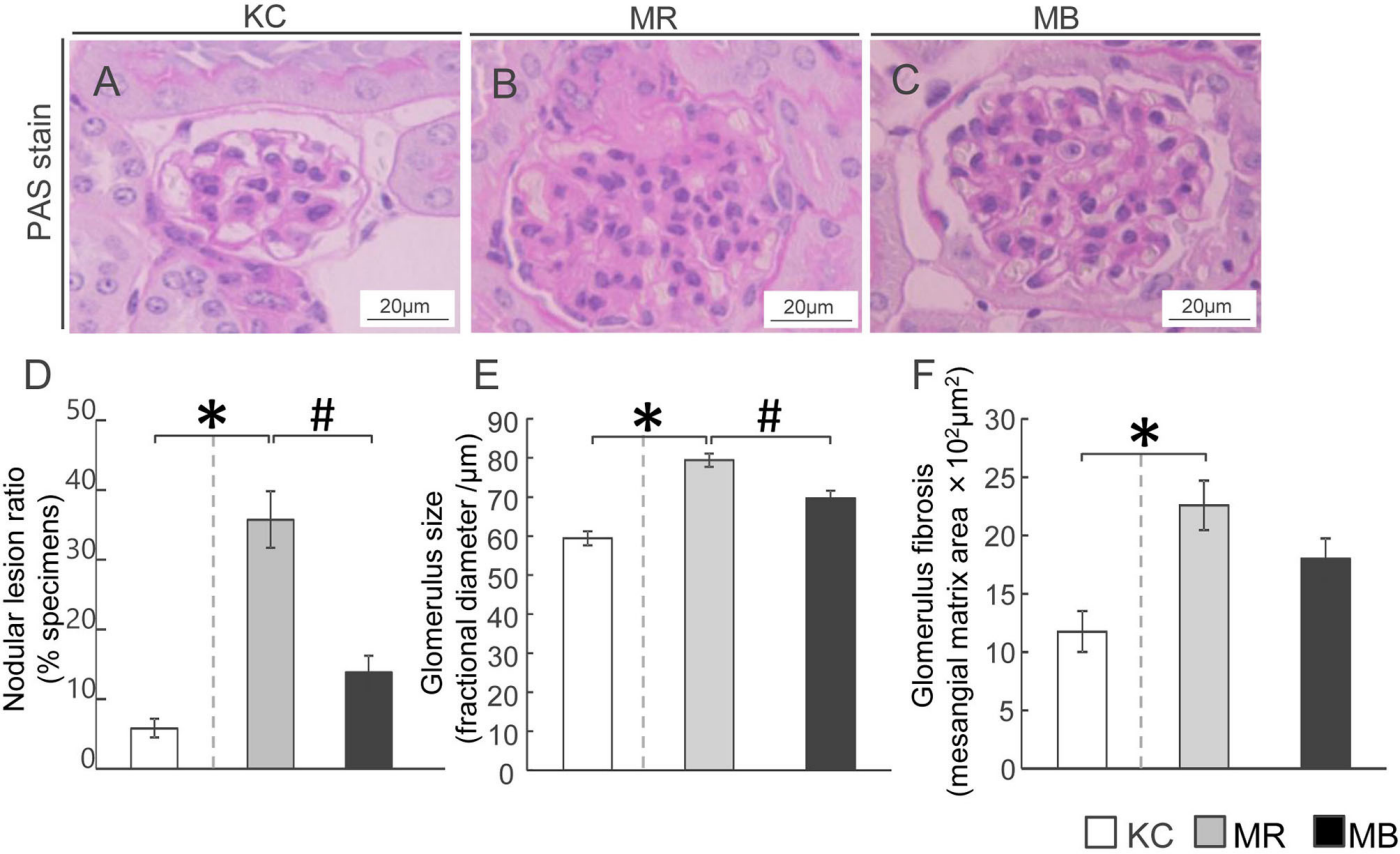
Histopathology showing fibrosis and pathogenesis in the kidneys of mice after 10 weeks of experimental diet intake. **(A–C)** Glomerular fibrosis observed using Periodic acid–Schiff staining (scale bar, 20 µm) in mice from the three groups. **(D–F)** Quantification of the nodular lesion ratio **(D)**, glomerular size **(E)**, and fibrotic area **(F)**. MB, minimum essential medium (MEM) mice on a barley diet; MR, MEM mice on a rice diet; KC, potassium simplex optimized medium control mice on a rice diet. Data are expressed as the mean ± SEM for 8–12 animals. Data were analyzed using Student’s *t*-test. *P < 0.05 compared with the KC group; ^#^ *P* < 0.05 compared with the MR group.

To ascertain whether the pathological characteristics and changes in the glomeruli of MEM mice were owing to the diabetic conditions, we performed PAS-staining (**Figures 2A–C**). The nodular lesion ratio, which is frequently measured in diabetic glomerular pathology, was higher in MR mice than in KC mice, whereas it was lower in MB mice than in MR mice (**Figure 2D**). The glomerular size, a measure of glomerular expansion, was greater in MR mice than in KC mice (**Figure 2E**) and lesser in MB mice than in MR mice. The glomerular fibrotic area, a measure of the mesangial matrix area, was larger in MR mice than in KC mice, but did not differ between MR mice and MB mice (**Figure 2F**). All images of PAS in the glomerulus of each group (**Supplementary Figures S4–S6**).

### Renal Arterial Changes in Diabetic MEM Mice

**Figure 3:**
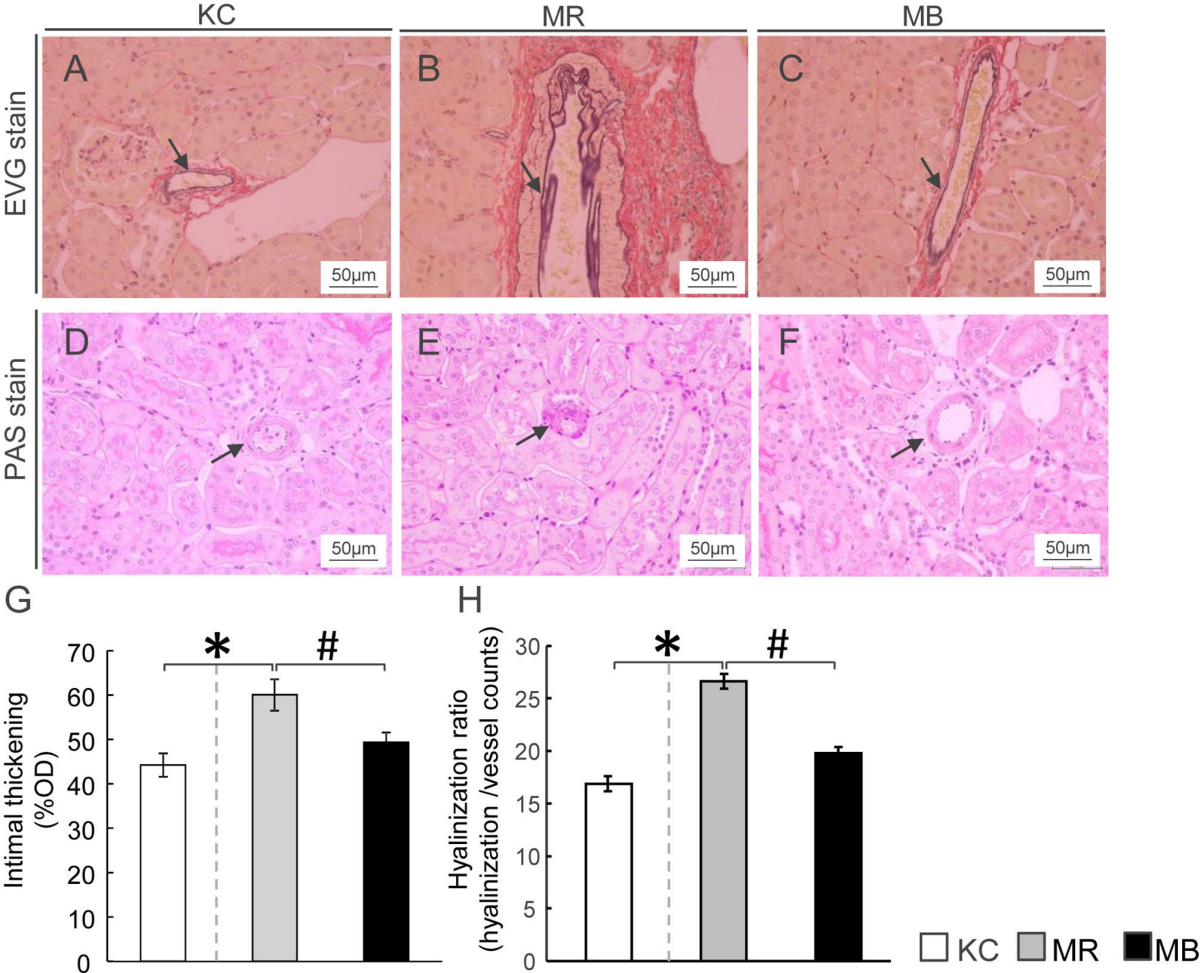
Histopathology showing renal arteriolar intimal thickening and hyalinosis in the kidneys of mice after 10 weeks of experimental diet intake. **(A–C)** Glomerular fibrosis observed using Elastica van Gieson staining (arrow) (scale bar, 50 µm) in mice from the three groups. **(D–F)** Renal arteriolar hyalinosis observed using Periodic acid–Schiff staining (arrow) (scale bar, 50 μm) in mice from the three groups. **(G, H)** Quantification of arteriosclerosis ratio (%OD) by fibro-intimal thickening/vascular media **(G)** and hyalinization of the intrarenal vasculature **(H)**. OD, outer diameter; MB, minimum essential medium (MEM) mice on a barley diet; MR, MEM mice on a rice diet; KC, potassium simplex optimized medium control mice on a rice diet. Data are expressed as the mean ± SEM for 8–12 animals. Data were analyzed using Student’s *t*-test. *P < 0.05 compared with the KC group; ^#^ *P* < 0.05 compared with the MR group.

To assess renal artery injury, we examined the intimal thickness and hyalinosis in the renal artery using EVG staining and PAS staining, respectively (**Figures 3A–F**). Intimal thickening, calculated as %OD, was higher in MR mice than in KC mice (**Figure 3G**), whereas it was lower in MB mice than in MR mice. The hyalinization ratio was higher in MR mice than in KC mice and lower in MB mice than in MR mice (**Figure 3H**). All images of PAS and EVG staining of the kidney of each group (**Supplementary Figures S4–S6**).

### Localization of Renal TGFB Protein

**Figure 4:**
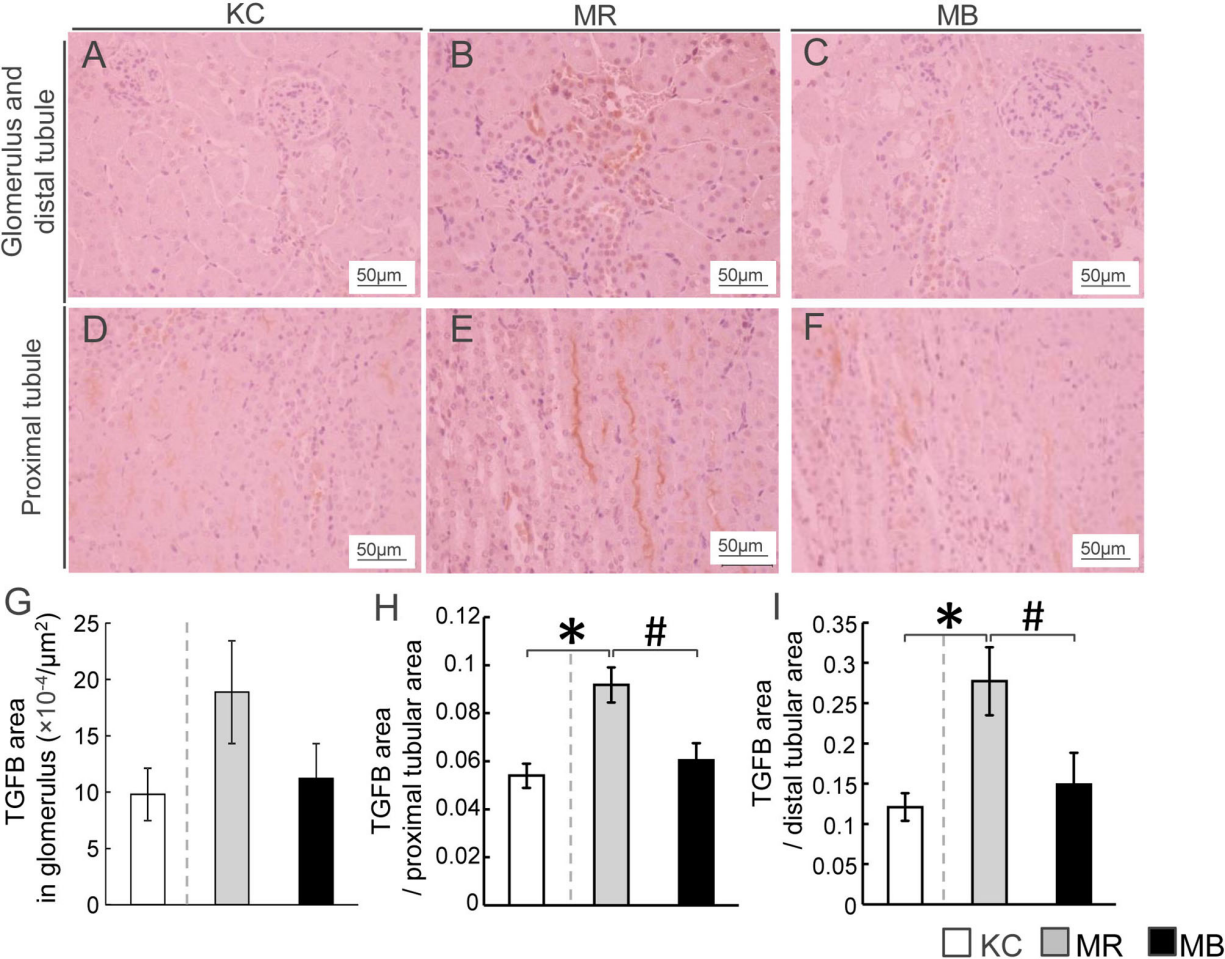
Histopathological evaluation by immunostaining for transforming growth factor beta (TGFB) and quantification of TGFB in the mice kidney tissues after 10 weeks of experimental diet intake. **(A–F)** Representative histological TGFB staining of sections of the glomerulus, distal tubule **(A–C)**, and proximal tubule **(D–F)** in mice from each of the three experimental groups (TGFB-positive areas are stained orange; scale bar, 50 µm). Quantification of the TGFB-positive area in the glomerulus **(G)**, proximal renal tubules **(H)**, and distal renal tubules **(I)**. MB, minimum essential medium (MEM) mice on a barley diet; MR, MEM mice on a rice diet; KC, potassium simplex optimized medium control mice on a rice diet. Data are expressed as the mean ± SEM for 8–12 animals. Data were analyzed using Student’s *t*-test. *P < 0.05 compared with the KC group; ^#^ *P* < 0.05 compared with the MR group.

We performed immunostaining to evaluate renal TGFB protein distribution, including that in the glomerulus and distal and proximal tubules (**Figures 4A–F**). The areas showing TGFB expression in the proximal and distal tubules were larger in MR mice than in KC or MB mice (**Figures 4H, I**). However, the differences in the glomerular TGFB expression levels were not significant between MR and KC mice or MB mice (*P*=0.16, *P*=0.21, respectively) (**Figure 4G**). All images of TGFB staining in the glomerulus and proximal/distal tuble of the kidney of each group (**Supplementary Figure S7–S9**). The TGFB protein level in total kidney did not differ between MR and KC mice or between MR and MB mice. (**Supplementary Figure S10**). There was no differences of the mRNA expression levels of inflammation cytokines between KC and MR, and between MR and MB in the kidney (**Supplementary Figure S11**). 

## Discussion

In the present study, we provided first evidence that MEM mice developed from two-cell-stage embryos cultured *in vitro* in αMEM exhibited pancreatic exhaustion and glomerulosclerosis, in contrast to control mice developed from embryos cultured in normal KSOM. Furthermore, the administration of dietary barley for 10 weeks after the mice reached an adult stage ameliorated these pathologies.

We observed a higher incidence of typical DKD pathology—nodular lesions and glomerular hypertrophy—in MEM mice that were fed a rice-based diet than in control KSOM mice. In this study, we did not observe microalbuminuria in MEM mice (data not shown), and the plasma phosphorus concentration did not differ between MEM mice fed a rice-based diet and control KSOM mice. The appearance of renal efferent arteriosclerosis in patients with T2DM is frequently associated with microalbuminuria, a marker of early-stage DKD. Thus, MEM mice at the stage evaluated in this study can be considered an animal model of DKD with similar pathology to patients with an earlier stage of DKD, although it remains unclear whether the continuous feeding of a high-fat, high-sugar diet to MEM mice causes DKD progression. Recent clinical studies on DKD suggest that the assessment of DKD development based on urine microalbumin levels is limited because progressive kidney dysfunction (eGFR < 60 mL/min/1.73 m^2^) is frequently observed in patients with T2DM with normoalbuminuria. The symptom is referred to as normoalbuminuric DKD (NADKD) or nonalbuminuric diabetic nephropathy (28, 29). There are no reports establishing NADKD animal models with severe renal damage similar to that observed in patients with NADKD. Further works should be examined whether animal models including our MEM mice models reflect human T2DM/DKD pathology by measuring biomarkers in human such as GFR or proteinuria. Additionally, only a few reports have shown that the symptoms in gene mutation-inducible or reagen-inducible DKD animals improve in response to dietary interventions. One study reported that the administration of epigallocatechin gallate (EGCg), a type of green tea extract, to *db/db* mice for 8 weeks reduced the mesangial matrix index by 34%, leading to glomerular dysfunction, in contrast to that in *db/db* mice that did not receive EGCg (30). In the present study, the administration of a barley diet for 10 weeks in adult MEM mice reduced the glomerular and nodular lesions as well as the renal arteriolar lesions. Furthermore, barley intake for only 10 weeks reduced the glomerular lesion ratio by 50%, intimal thickening by 15%, and hyalinosis by 27%. Taken together, MEM mice may be considered NADKD animal models with a similar pathology to patients with DKD, with nodular sclerosis in the glomeruli, and the DKD pathology can be attenuated by dietary interventions.

In this study, we found that the renal arteriolar hyalinosis rate and intimal thickening of the renal arteriola, both of which indicate renal efferent arteriosclerosis (31), were higher in MEM mice fed a rice-based diet than in control KSOM mice. Renal vascular lesions in DKD are linked to renal efferent arteriosclerosis, in which there is an increase of ECM in the interior of renal vascular vessels, which is related to hypertension. Hypertension is one of the strongest risk factors for renal arteriolosclerosis. The elevation of blood pressure induces endothelial collapse and subsequent endothelial cell proliferation for the repair of the renal arteriola, which leads to the development of renal efferent arteriosclerosis (32). Reportedly, greater renal arteriolar hyaline degeneration as well as mesangial matrix proliferation and glomerular fibrosis were observed in patients with NADKD with exacerbated eGFR (<60 mL/min/1.73 m^2^) than in patients with normal eGFR NADKD, but there was no difference in the systolic/diastolic blood pressure between patients with NADKD with normal/exacerbated eGFR (33). In representative conventional T2DM models, such as *ob/ob* mice and *db/db* mice, renal efferent arteriosclerosis as well as glomerular lesions were observed in endothelial nitric oxide synthase (eNOS)-deficient *db/db* mice (34) and -*ob/ob* mice of the BTBR strain (35). eNOS expression in the endothelium is associated with the reduction of blood pressure *via* the relaxation of blood vessels (36). These results indicate that hypertension with diabetes is associated with the development of renal efferent arteriosclerosis. Therefore, renal efferent arteriosclerosis in MEM mice may be caused by hypertension, and the improvement of renal efferent arteriosclerosis in MEM mice by barley intake may require the reduction of hypertension. However, in this study, we did not measure the blood pressure of MEM mice. Reportedly, rat offspring that underwent protein restriction (9% casein) during the fetal period showed reduced nephron number (37). In a human cohort study, participants with a low birth weight had a higher incidence of hypertension at 36 years of age compared to participants with normal birth weight (38), and participants born to mothers who experienced severe famine during early gestation had a higher incidence of hypertension and kidney disease at 58 years of age compared with those born to mothers who did not experience famine during gestation (39). It remains unclear how renal efferent arteriosclerosis is ameliorated by barley intake. A placebo-controlled randomized trial called Study TO Prevent Non-Insulin-Dependent Diabetes Mellitus (STOP-NIDDM) reported that treatment with acarbose, an α-glucosidase inhibitor, in mildly impaired glucose tolerance reduced not only the risk of T2DM progression (36%) (40) but also a develop risk of hypertension (34%) and cardiovascular disease (49%) (41). In addition, treatment with the α-glucosidase inhibitor miglitol (42) and barley (23) lowered the expression of inflammatory cytokines, such as interleukin 1 beta and tumor necrosis factor alpha, and integrins, such as CD11s in peripheral leukocytes of rodents with diabetes. Further investigation is needed to confirm the association between renal arteriolosclerosis and hypertension by blood pressure measurement in MEM mice.

In this study, we found that the number of insulin-positive β cells was lower and the number of fibrotic-areas was higher in the pancreas of MEM mice fed a rice-based diet than in KSOM control mice. Further, the reduction in the number of insulin-positive β cells and expansion of fibrotic areas in the pancreas of MEM mice was ameliorated by barley intake for 10 weeks in the adult stage. Therefore, pancreatic exhaustion in MEM mice was suppressed by barley administration for 10 weeks. A previous study on a T2DM animal model–the Goto-Kakizaki (GK) rat–demonstrated that 8-week treatment with miglitol, which is an α-glucosidase inhibitor, suppressed postprandial hyperglycemia, in contrast to that in non-treated GK control rats, but it did not improve the insulin secretion capacity and pancreatic exhaustion (43). Another study reported that the miglitol treatment of OLETF rats improved pancreatic exhaustion, but improvement was observed only after treatment for more than 1 year (65 weeks) (44). Additionally, dietary supplementation with acarbose, an α-glucosidase inhibitor, in *db/db* mice for 4 weeks moderately improved the insulin secretion capacity compared with that in *db/db* mice that did not receive the supplement. However, considerably high doses of acarbose (such as 9 g/kg/day) were needed, whereas patients with T2DM are administered doses of 150−300 mg/day (45). Therefore, the MEM mouse is an effective T2DM model that can be used to assess dietary factors and/or drugs to improve T2DM and DKD. However, it should be confirmed whether the T2DM and DKD pathologies in MEM mice are in fact improved by other dietary factors and drugs. Of note, the body weight did not differ significantly between MEM mice fed a rice-based diet and KSOM control mice, whereas food intake was greater in MEM mice fed a rice-based diet than in KSOM control mice. MEM mice show characteristic hyperglycemia with a slightly higher weight and reduced capacity of insulin secretion from the pancreas in spite of overeating, similar to lean Asian patients with T2DM, as shown in a previous study (20). In future studies, it should be investigated whether the capacity of insulin secretion is lower from a prior to development of T2DM/DKD in MEM mice. In addition, it should be examined whether MEM mice can develop T2DM/DKD even when pair-feeding is performed for equal consumption between MEM and KSOM mice.

In the present study, we demonstrated that barley intake improved DKD pathologies, including tuberous sclerosis and renal arteriolar changes, in MEM mice. It was previously reported that barley intake decreased the blood glucose levels 30 min after the meal in healthy participants, suggesting that barley intake can decrease the postprandial blood glucose concentration (46). We previously performed OGTT to assess the glucose tolerance of MEM mice, but the assay could not assess the postprandial blood glucose levels after barley intake in diurnal variation (23). Therefore, the improvement in DKD pathology in MEM mice after barley intake could be attributed to the reduction of postprandial hyperglycemia by barley. Another potential contributor is the prebiotic effect of β-glucan, which is an abundant soluble dietary fiber present in barley. Indeed, it was reported that the daily oral administration of β-glucan (80% purity) at 1 g/kg body weight/day in specific pathogen-free mice fed a 12-week high-fat (60% kcal) diet altered the intestinal bacterial flora in present in feces, although the particular role of the bacterial flora is not fully understood (47). Barley intake for 8 weeks increased the proliferation of probiotic *Lactobacillus* strains, such as *Prevotella*, *Lactobacillus*, and the fiber-degrader S24-7 (*Candidatus Homeothermaceae*) in obese *db/db* mice compared to that in lean *db/m* control or obese *db/db* mice fed a control diet (48). Further studies are needed to examine whether the intestinal bacterial flora altered by barley intake can improve DKD in MEM mice.

The components of the αMEM responsible for the development of T2DM/DKD pathologies in MEM mice remain unknown, even though the components, such as non-essential amino acids and vitamins, differ between KSOM and αMEM (**Supplementary Table S1**). In human clinical practice, αMEM is not used in the *in vitro* culture of embryos in *in vitro* fertilization (IVF), and media with relatively simple ingredients, similar to KSOM, are used. A study demonstrated that in spite of the same body-mass index, at puberty, children born *via* IVF had higher fasting blood glucose levels, systolic and diastolic blood pressure (49), and peripheral fat mass (50) than children born by spontaneous delivery. In addition, it was reported that children born *via* IVF have a higher risk of low birth weight (<2,500 g) and cardiovascular hospitalization incidence until 18 years compared with children born *via* spontaneous delivery (51) This shows that *in vitro* embryo culture in the early embryo stage may affect health risks, such as that for T2DM/DKD, after birth, and the optimal medium for IVF is still under investigation in human clinical practice. Further studies are needed to identify the components of αMEM that influence the T2DM/DKD pathology in MEM mice to optimize the culture media for assisted reproductive technology (ART) with a low health hazard risk and to determine the mechanism underlying αMEM exposure-induced DKD pathogenesis at the two-cell embryo stage. In addition, the IVF medium used in human clinical practice should be optimized by evaluating T2DM and DKD development in mice that have developed from embryos subjected to *in vitro* culture in the medium. However, the conditions in the mice models are different from those in human disease; therefore, further studies are needed to examine whether the different methods used in ART induce metabolic disorders in mice and humans.

Of note, the glomerular distribution of TGFB protein, a strong risk factor for the development of glomerulosclerosis by mesangial matrix expansion (52), did not differ between MEM mice fed a rice-based diet and KSOM control mice. It has also been reported that *db/db* mice, which exhibit severe non-fasting hyperglycemia (34.8 ± 6.3 mM (mean ± SEM) at 25 weeks of age) (53), expressed TGFB and showed mesangial matrix expansion in the glomerulus. Therefore, MEM mice developed early-stage and non-fulminant DKD. Interestingly, we found that TGFB distribution in the proximal/distal renal tubules was higher in MEM mice fed a rice-based diet than in KSOM control mice. In addition, after MEM mice reached an adult stage, barley intake suppressed TGFB expression in the renal tubules. Kidney sections have been studied to show that TGFB expression is relatively higher in the renal tubules than in the glomerulus in patients with T2DM (54). The kidney contains diverse cell types (including the cells in the glomerulus and proximal/distal tubules) with varied functions. TGFB is reportedly expressed in renal tubules as well as in the glomerulus during the development of nephropathy (55); however, the roles of TGFB in renal tubular dysfunction remain unclear. The results of the present study suggest that a higher TGFB expression in the renal tubules of MEM mice may indicate renal tubular dysfunction. Further studies are required to identify the stage of DKD in MEM mice, to measure TGFB expression in the tubules or glomeruli of MEM mice and patients with DKD, and to determine the roles of TGFB in renal tubular dysfunction. In addition, with the progression of DKD in T2DM, MEM mice from the pre-DKD stage to late-DKD stage should be assessed to confirm whether MEM mice models show pathologies similar to those observed in human T2DM/DKD.

In conclusion, T2DM MEM mice formed from two-cell stage embryos cultured *in vitro* in α-MEM developed DKD, and barley intake after birth ameliorated the DKD pathology in these mice.

## Data Availability Statement

The datasets presented in this study can be found in online repositories. The names of the repository/repositories and accession number(s) can be found in the article/**Supplementary Material**.

## Ethics Statement

The animal study was reviewed and approved by the Ethics Committee of the University of Yamanashi (approval number A30-24). Written informed consent was obtained from the owners for the participation of their animals in this study.

## Author Contributions

SK and KM conceptualized and developed the MEM mice, experiments, and analytical approaches. SI, MK, and TN conducted the experiments. MK and TN contributed to MEM mice development and sample preparation. SI performed histological analyses, biochemical determination, mRNA analysis, western blotting, and statistical analyses. KU contributed assessment of kidney histological analyses and the discussion. SI and KM wrote the paper with contributions from all authors. YF, KU, SK, and KM performed the critical review of the manuscript.

## Funding

This work was supported by the Mishima Kaiun Memorial Foundation, a Grant-in-Aid for Scientific Research from the Ministry of Education, Culture, Sports, Science, and Technology of Japan [20H04103], the Adaptable and Seamless Technology Transfer Program through target-driven R&D (A-STEP) of the Japan Science and Technology Agency (JST), and the Council of Japan Barley Foods Promotion.

## Conflict of Interest

Author TN was employed by company Kiwa Laboratory Animals Co., Ltd.

The remaining authors declare that the research was conducted in the absence of any commercial or financial relationships that could be construed as a potential conflict of interest.

## Publisher’s Note

All claims expressed in this article are solely those of the authors and do not necessarily represent those of their affiliated organizations, or those of the publisher, the editors and the reviewers. Any product that may be evaluated in this article, or claim that may be made by its manufacturer, is not guaranteed or endorsed by the publisher.

## Abbreviations

αMEM, α minimum essential medium; ART, assisted reproductive technology; DKD, diabetic kidney disease; DOHaD, Developmental Origins of Health and Disease; ECM, extracellular matrix; EGCg, epigallocatechin gallate; eGFR, estimated glomerular filtration rate; eNOS, endothelial nitric oxide synthase; EVG, Elastica van Gieson; GFR, glomerular filtration rate; GK rat, Goto-Kakizaki rat; HE, hematoxylin-eosin stain; ICR, Institute of Cancer Research; IVF, *in vitro* fertilization; KC, mice developed from embryos cultured in KSOM and subsequently fed a rice-based diet; KSOM, potassium simplex-optimized medium; MDA, malondialdehyde; MEM, minimum essential medium; MB, mice developed from embryos cultured in MEM and fed a barley-based diet; MR, mice developed from embryos cultured in MEM and fed a rice-based diet; MT, Masson’s trichrome; NADKD, normoalbuminuric diabetic kidney disease; NADPH, nicotinamide adenine dinucleotide phosphate; OD, outer diameter; OGTT, oral glucose tolerance test; PAS, Periodic acid-Schiff; SEM, standard error of the mean; TGFB, transforming growth factor beta; T2DM, type 2 diabetes mellitus; 8-hydroxydeoxyguanosine, 8-OHdG.
