# Supplementary material for: Development of the Diabetic Kidney Disease Mouse Model Culturing Embryos in α-Minimum Essential Medium In Vitro, and Feeding Barley Diet Attenuated the Pathology
Source: Front Endocrinol (Lausanne). 2021 Nov 2;12:746838. doi: 10.3389/fendo.2021.746838 (PMC8634848; doi:10.3389/fendo.2021.746838)
Supplement: Supplementary file 1 [file DataSheet_1.doc]

**Supplemental methods**

*Quantitative real-time RT-PCR (qRT-PCR)*

Total RNA from kidney was isolated using a formula based on the acid guanidinium thiocyanate-phenol-​chloroform extraction (AGPC) method as described previously [1]. In brief, tissue was homogenized with guanidine thiocyanate as protein denaturant and RNA was extracted with phenol / chloroform. Subsequently, cDNA from extracted RNAs were generated by reverse transcription using SuperScript III reverse transcriptase (Thermo Fisher Scientific Inc), following the manufacturer’s protocol. cDNA was amplified using LightCycler 480 SYBR Green Ⅰ Master and LightCycler System (Roche Diagnostics K.K.). Table S3 shows the used primer sequences for mouse. Data were normalized to a housekeeping gene (cytochrome c-1, *Cyc1*), following the delta-delta Ct (threshold cycle) method [2]: 2(Ct *Cyc1 −* Ct target gene) for qRT PCR results as described previously [3] A sample of RNA in the kidney of MEM mice fed a control diet group was not amplified well, therefore, we removed the qRT-PCR data of the mouse sample (n=11).

*Western blotting*

Kidney tissue was lysed with RIPA and the protein extracts were electrophoresed by the SDS-polyacrylamide gel electrophoresis (PAGE) method as described previously [4]. Briefly, protein levels of tissue extract in RIPA were determined by the Lowry method [5], then lysates with 48 μg of protein were incubated at 65°C for 20 min, subsequently subjected to SDS-PAGE to separate. Wet-transfer was then performed from 14% gel containing 100mM EDTA to polyvinylidene fluoride (PVDF) membranes (Immobilon-P, EMD Millipore Corporation, Billerica, MA, USA) in tris/glycine transfer buffer at 60 V for 30 min. Western blot analysis was performed with a primary antibody against all subtypes of TGFB (rabbit polyclonal antibody, #3711, Cell Signaling Technology, Beverly, MA, USA) in enhancer solution 1 (Can get signal; TOYOBO CO., LTD., Osaka, Japan) according to manufacturers’ instructs after blocked with for 1 h in 10% skim milk in PBS containing 0.5 M NaCl and 0.05% Tween 20 (PBS-T). Blots were then incubated with the secondary antibodies anti-rabbit IgG (RPN1004, biotinylated species-specific whole antibody, GE Healthcare, Little Chalfont, UK) in enhancer solution 2 according to manufacturers’ instructs. The membranes were then incubated with horseradish peroxidase-linked anti-biotin antibody (1:2,000; #7075, Cell Signaling Technology) in enhancer solution 2 according to manufacturers’ instructs. Blots were detected using enhanced chemiluminescence (PerkinElmer, Waltham, MA, U.S.A.) and intensities were measured with scanning densitometry using ChemiDoc XRS Plus System (Bio-Rad Laboratories, Inc., Hercules, CA, USA). The membrane was stained with 0.125% Coomassie brilliant blue (Sigma-Aldrich, St. Louis, MO, USA) in 20% methanol and 10% acetic acid to normalize signal level by the total transferred protein level in each lane, as described previously [6]. The number of gel wells for SDS-PAGE was limited, thus the randomized lysates samples were used for analysis by RAND function of Microsoft Excel.

*Reference*

1. Chomczynski P, Sacchi N (1987) Single-step method of RNA isolation by acid guanidinium thiocyanate-phenol-chloroform extraction. Anal Biochem 162: 156-159.

2. Livak KJ, Schmittgen TD (2001) Analysis of relative gene expression data using real-time quantitative PCR and the 2(-Delta Delta C(T)) Method. Methods 25: 402-408.

3. Farahzadi R, Fathi E, Vietor I (2020) Mesenchymal Stem Cells Could Be Considered as a Candidate for Further Studies in Cell-Based Therapy of Alzheimer's Disease via Targeting the Signaling Pathways. ACS Chem Neurosci 11: 1424-1435.

4. Suzuki T, Muramatsu T, Morioka K, Goda T, Mochizuki K (2015) ChREBP binding and histone modifications modulate hepatic expression of the Fasn gene in a metabolic syndrome rat model. Nutrition 31: 877-883.

5. Lowry OH, Rosebrough NJ, Farr AL, Randall RJ (1951) Protein measurement with the Folin phenol reagent. J Biol Chem 193: 265-275.

6. Ishiyama S, Kimura M, Umihira N, Matsumoto S, Takahashi A, Nakagawa T, et al. (2021) Consumption of barley ameliorates the diabetic steatohepatitis and reduces the high transforming growth factor beta expression in mice grown in alpha-minimum essential medium in vitro as embryos. Biochem Biophys Rep 27: 101029.
