# Supplementary figures and images for: Development of the Diabetic Kidney Disease Mouse Model Culturing Embryos in α-Minimum Essential Medium In Vitro, and Feeding Barley Diet Attenuated the Pathology
Source: Front Endocrinol (Lausanne). 2021 Nov 2;12:746838. doi: 10.3389/fendo.2021.746838 (PMC8634848; doi:10.3389/fendo.2021.746838)

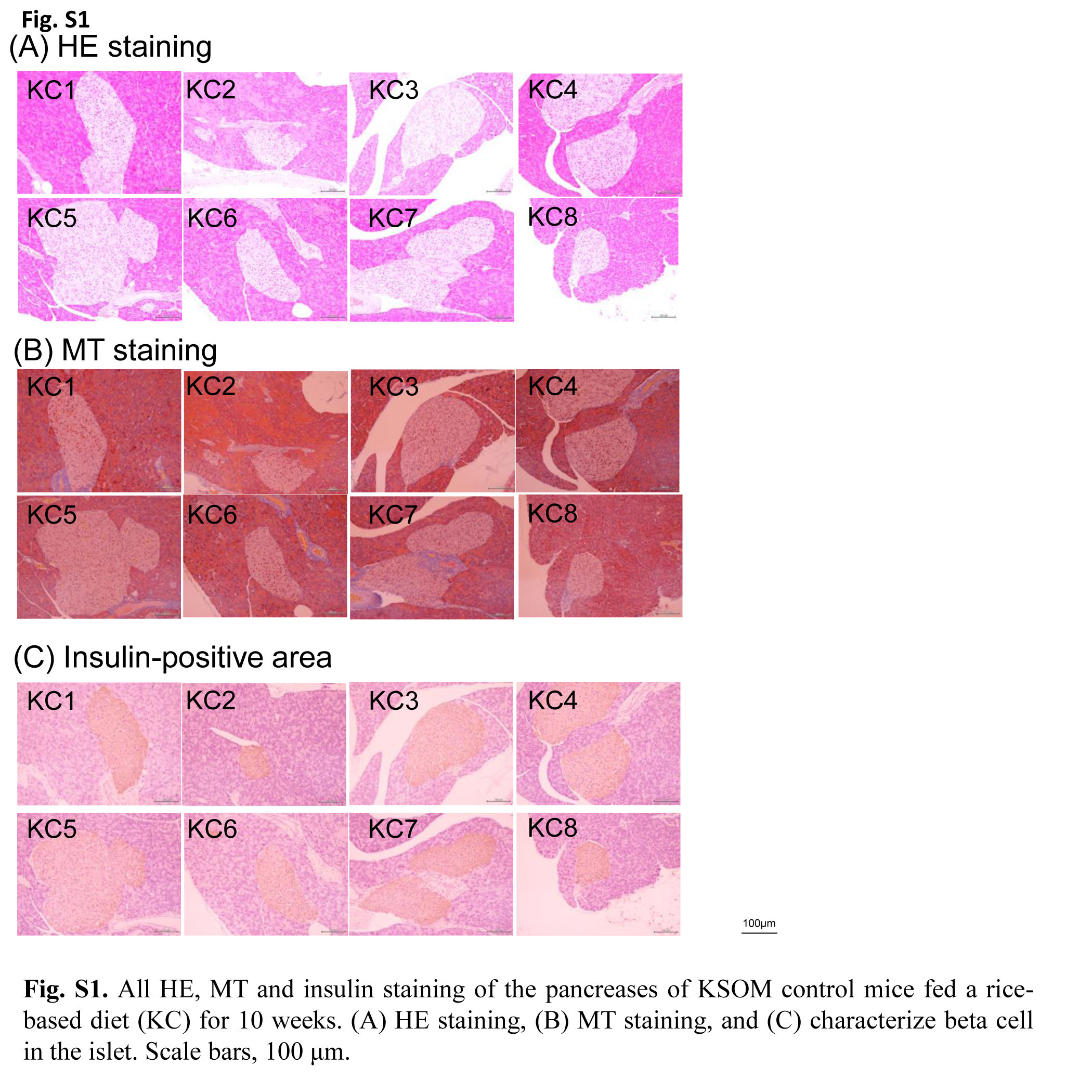

Supplement: Supplementary file 2 [file Image_1.jpeg]

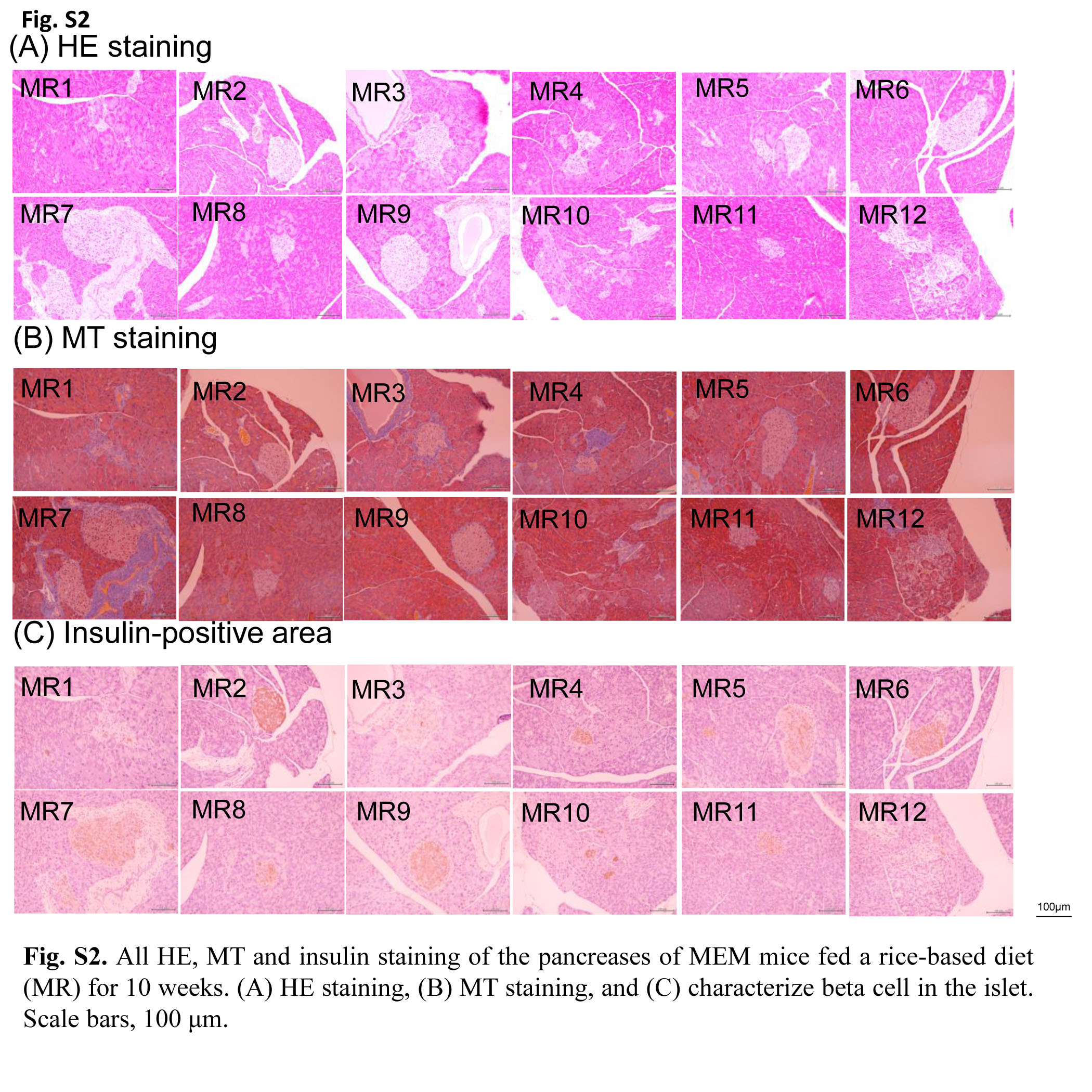

Supplement: Supplementary file 3 [file Image_2.jpeg]

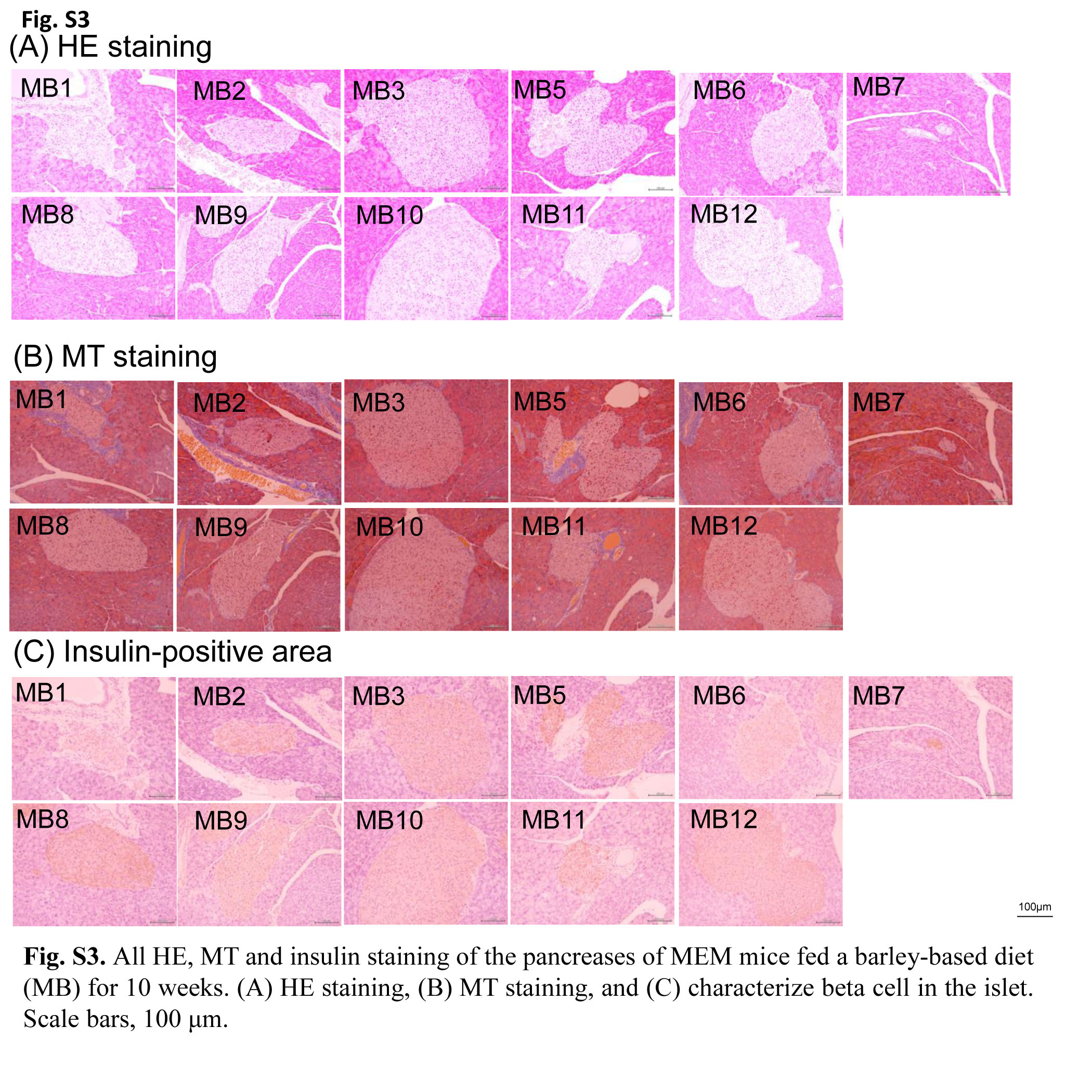

Supplement: Supplementary file 4 [file Image_3.jpeg]

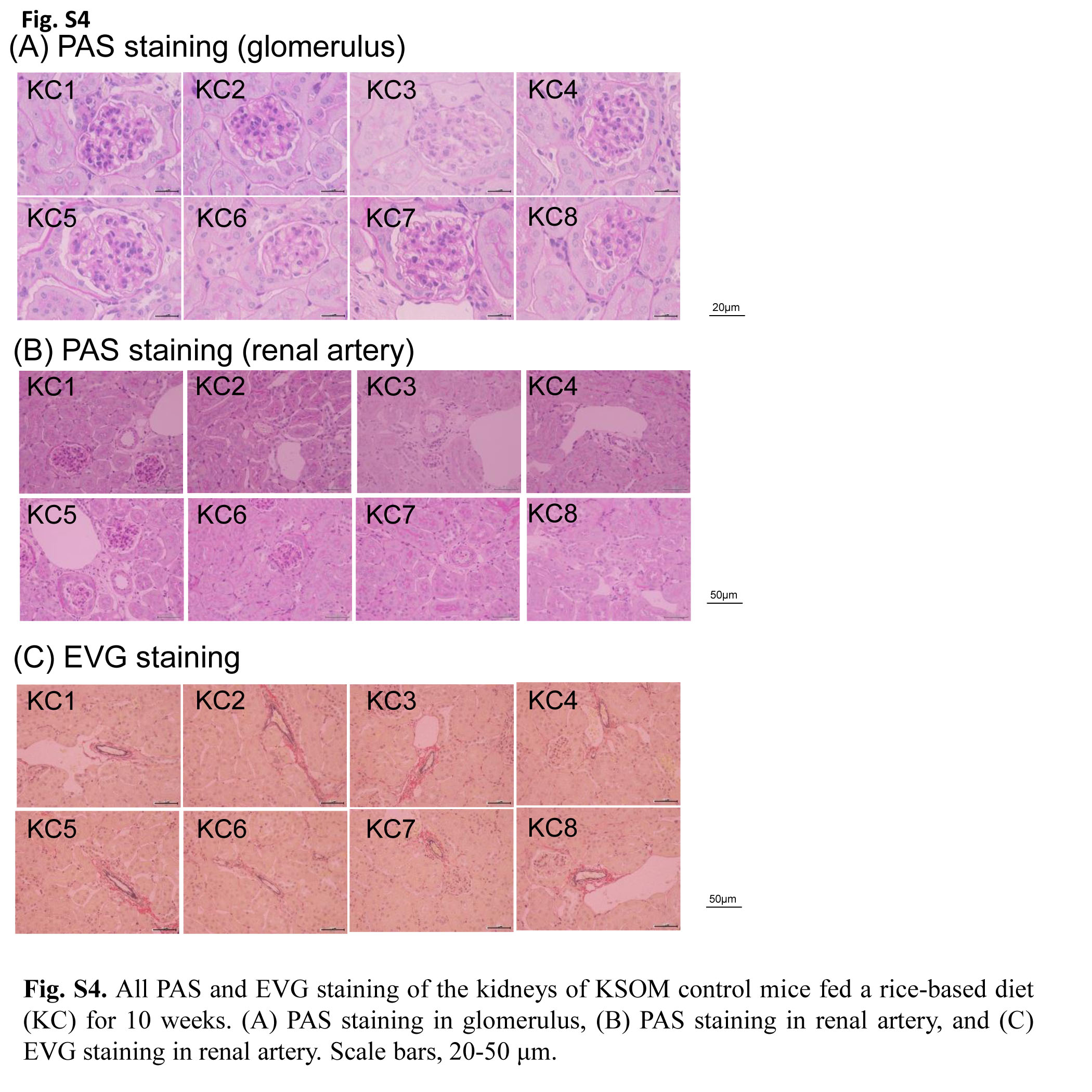

Supplement: Supplementary file 5 [file Image_4.jpeg]

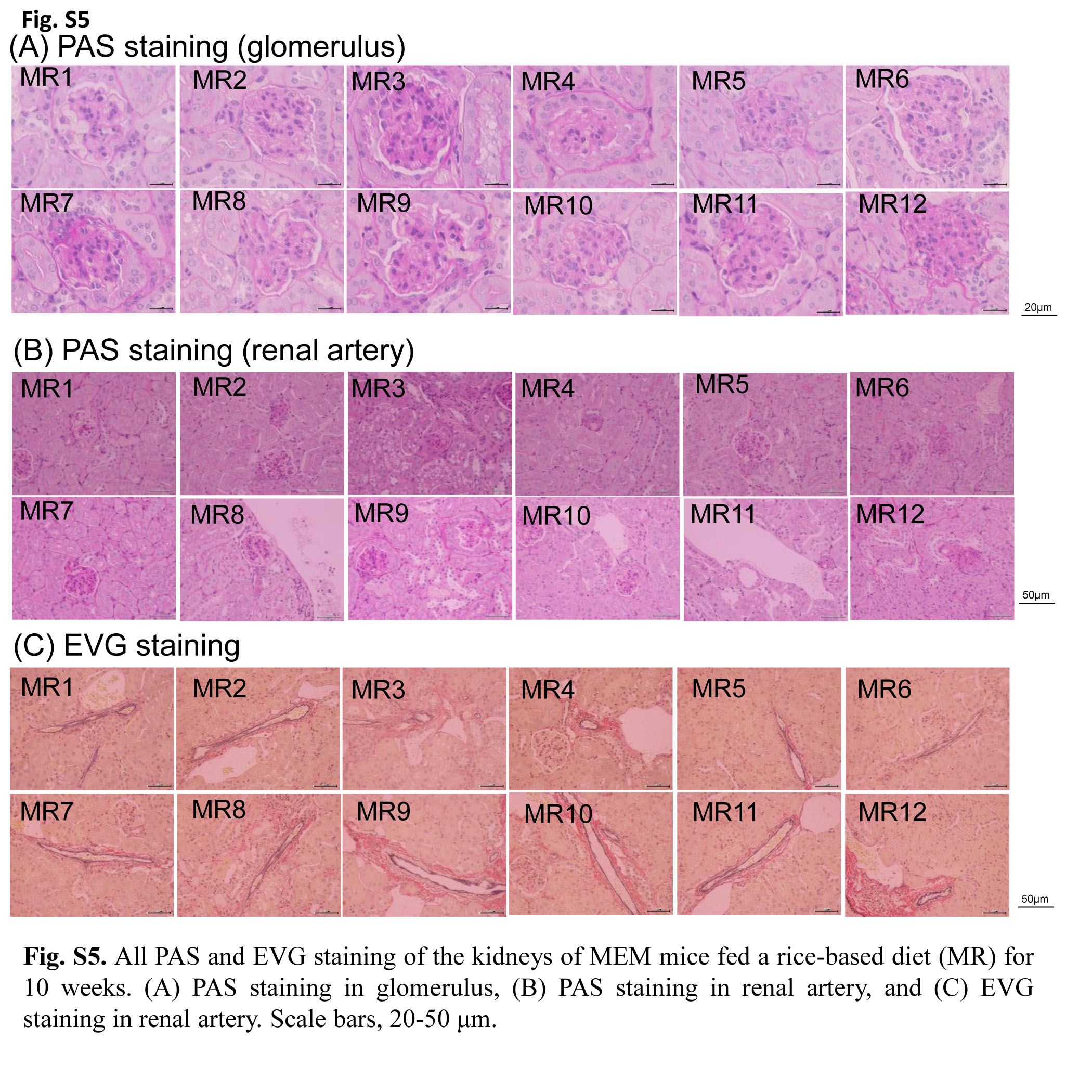

Supplement: Supplementary file 6 [file Image_5.jpeg]

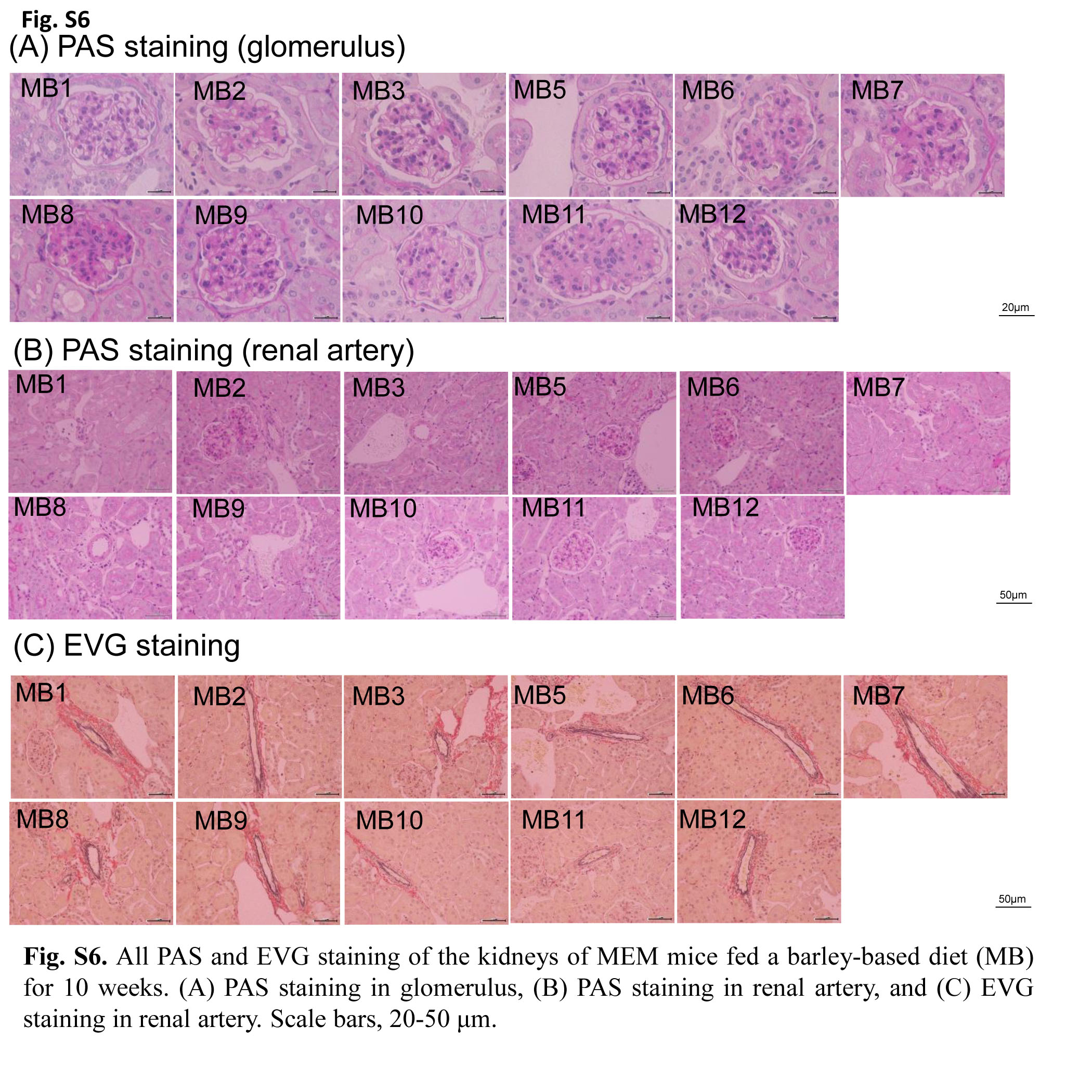

Supplement: Supplementary file 7 [file Image_6.jpeg]

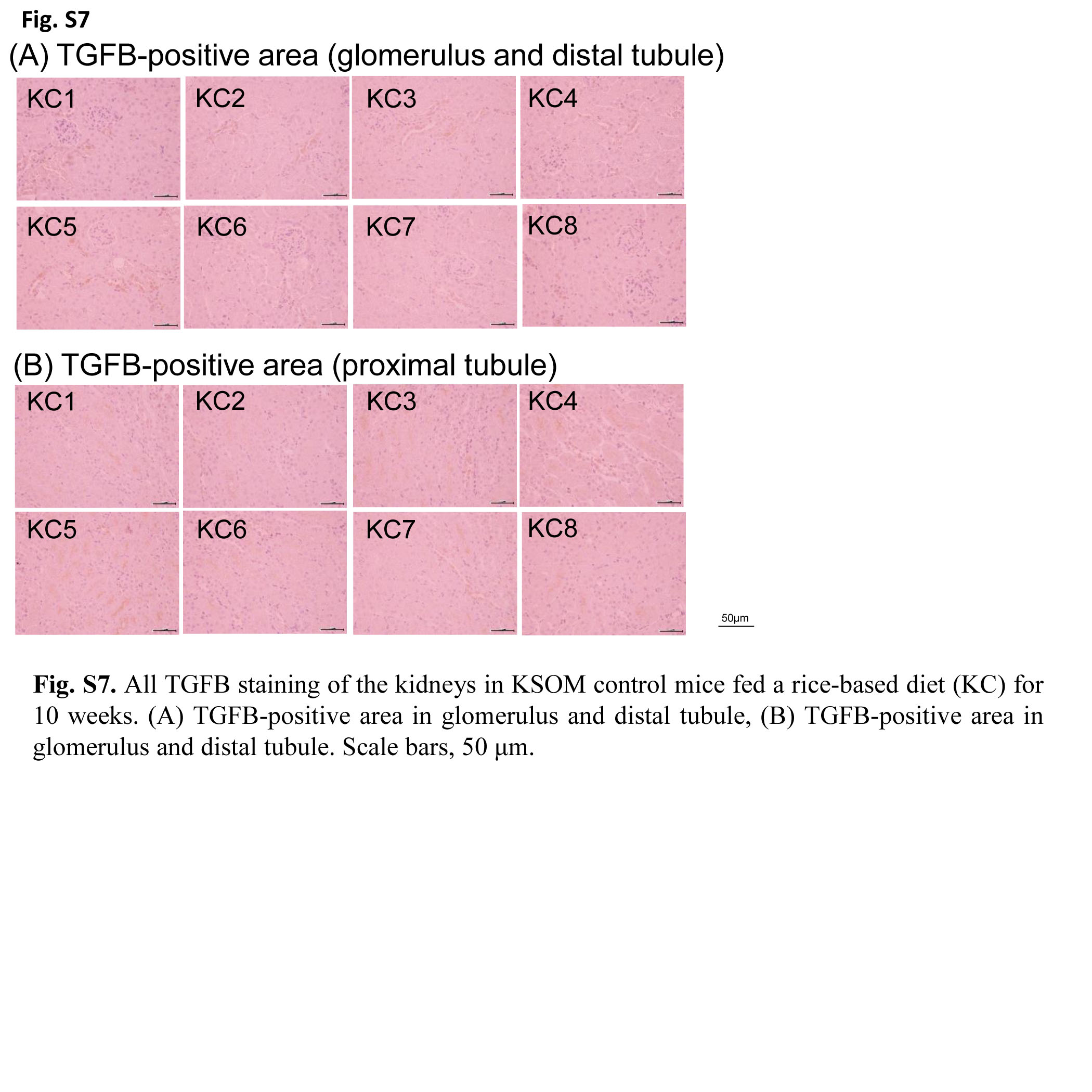

Supplement: Supplementary file 8 [file Image_7.jpeg]

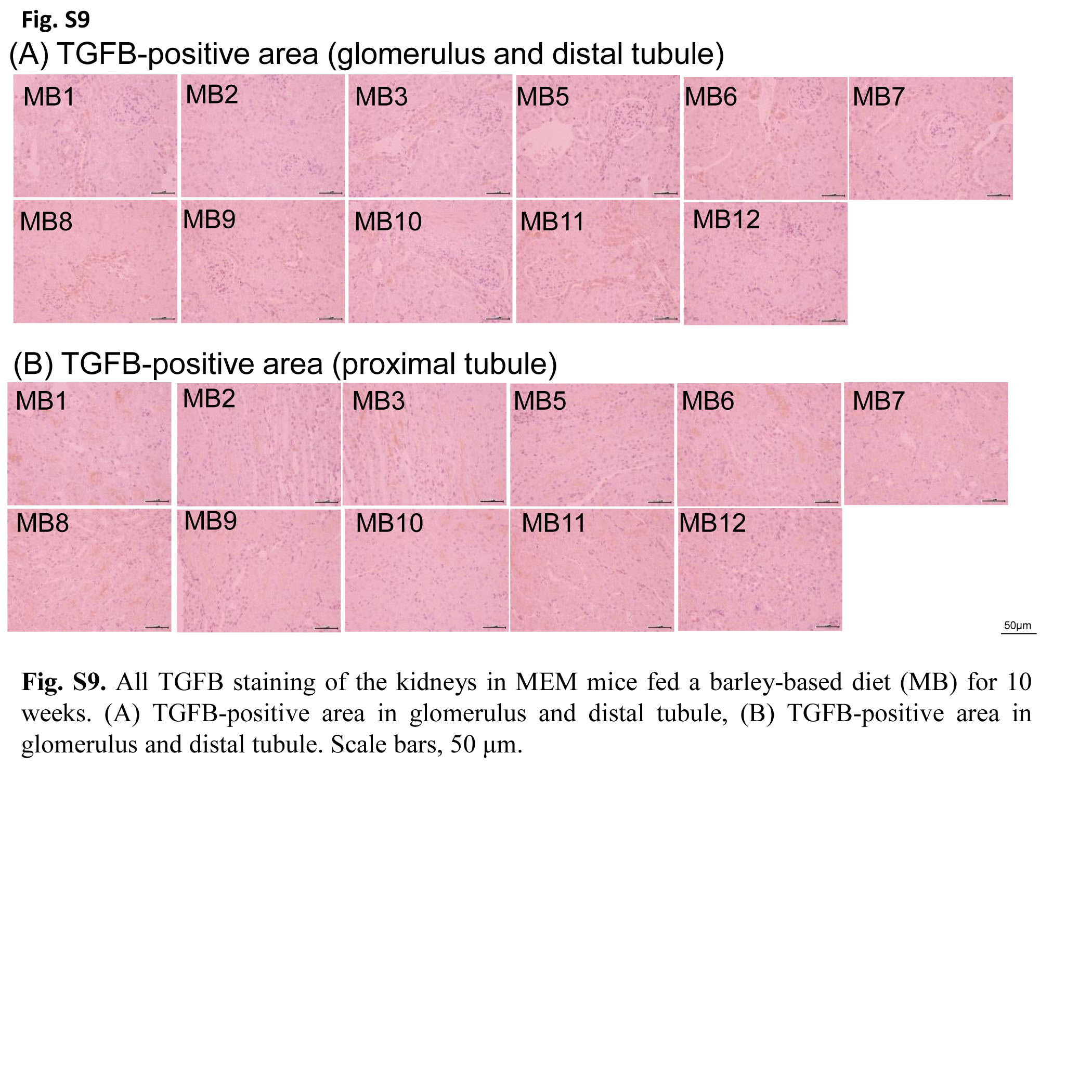

Supplement: Supplementary file 9 [file Image_9.jpeg]

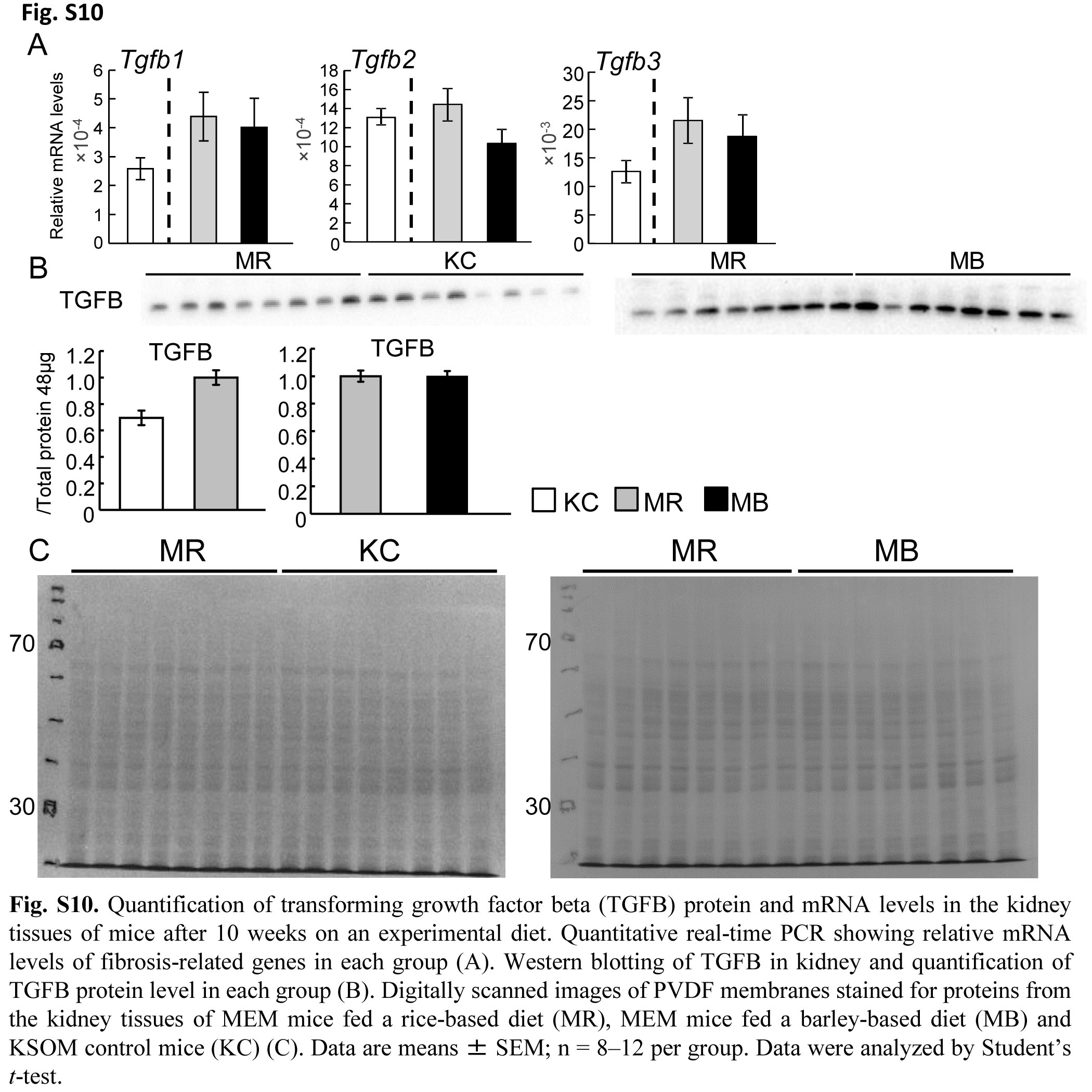

Supplement: Supplementary file 10 [file Image_10.jpg]

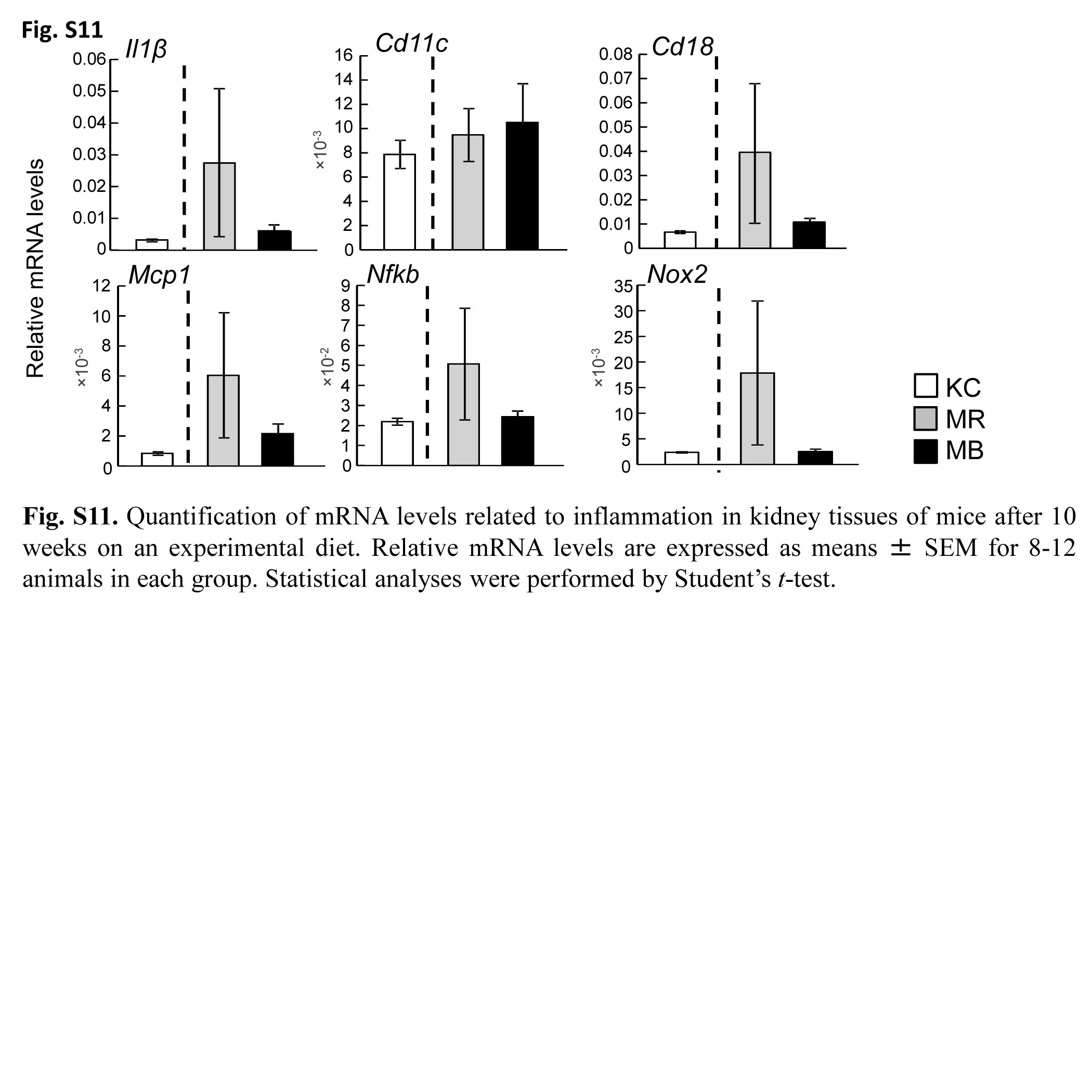

Supplement: Supplementary file 11 [file Image_11.jpg]
